# Supplementary material for: Living shorelines achieve functional equivalence to natural fringe marshes across multiple ecological metrics
Source: PeerJ. 2021 Aug 13;9:e11815. doi: 10.7717/peerj.11815 (PMC8366526; doi:10.7717/peerj.11815)
Supplement: Supplemental Information 5 [file peerj-09-11815-s005.pdf]

Supplemental Figures

Robert Isdell

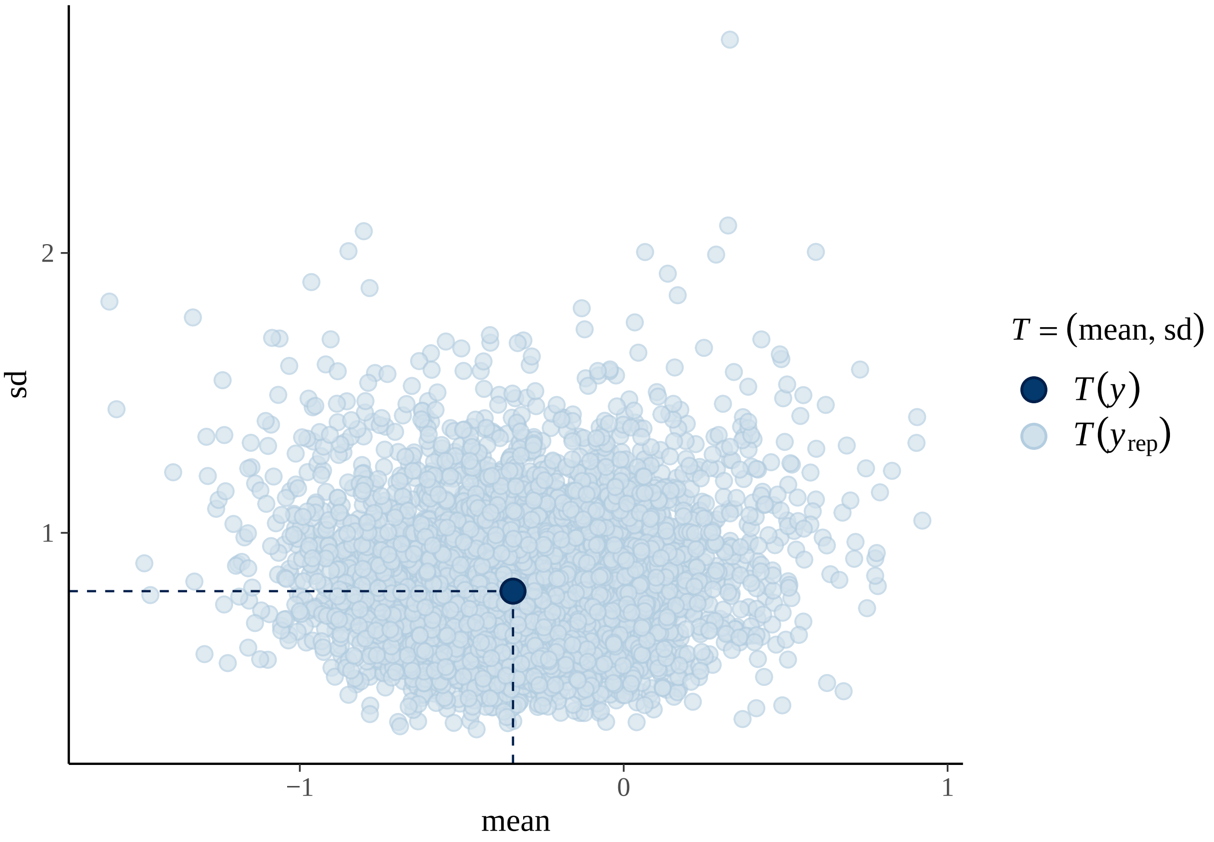

Figure S1: Posterior predictive check that the model adequately captures the parameters of the data. This figure is for the model that uses only the mussels from both the low marsh and the sill.

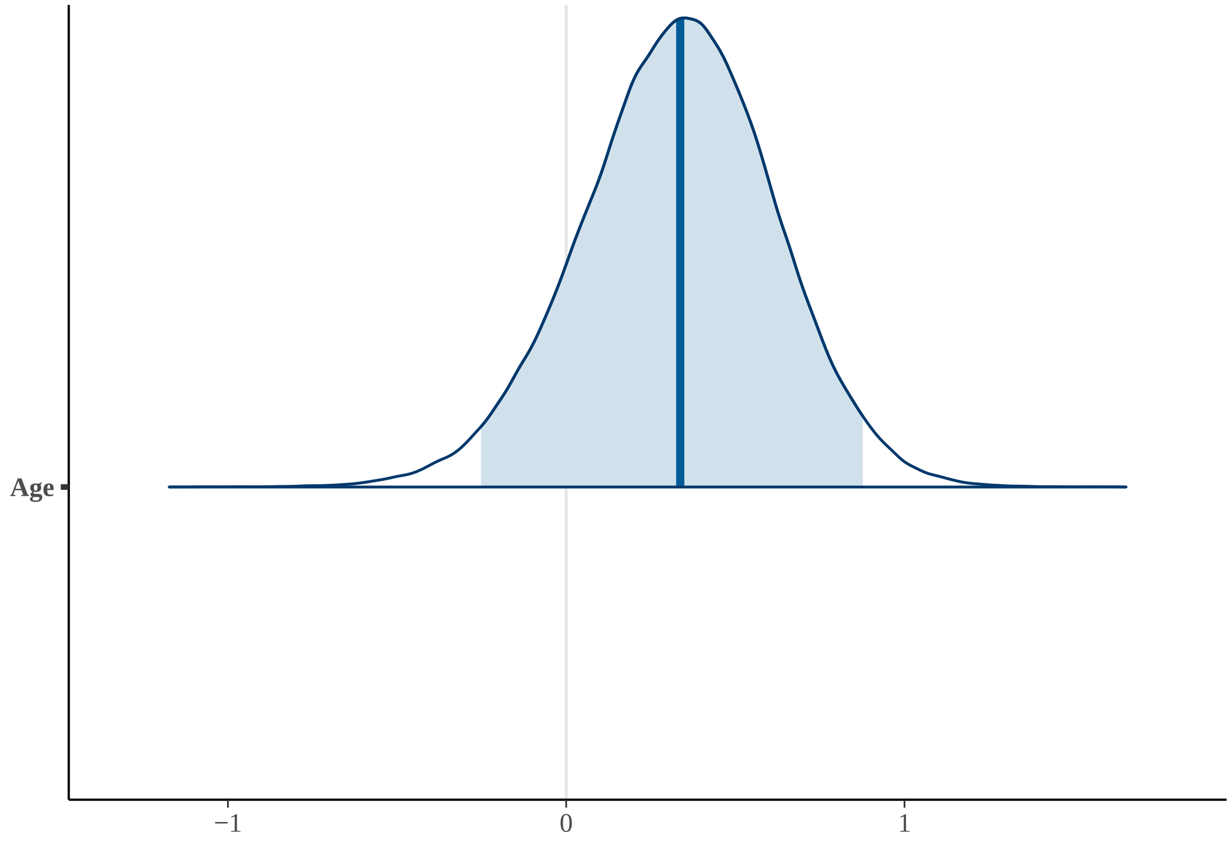

Figure S2: Posterior distribution of the living shoreline age coefficient from the model including mussels in the low marsh and on the sill.

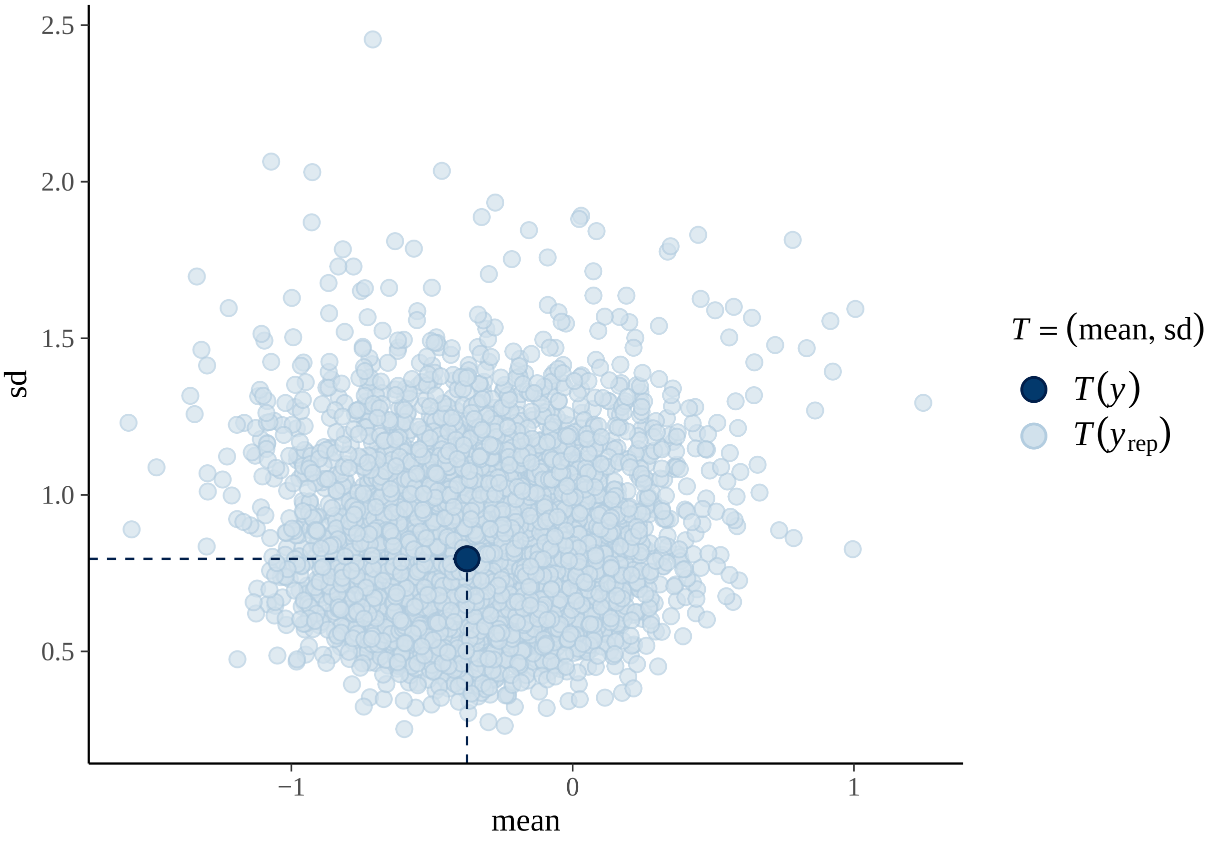

Figure S3: Posterior predictive check that the model adequately captures the parameters of the data. This figure is for the model that uses only the mussels from the low marsh and not the sill.

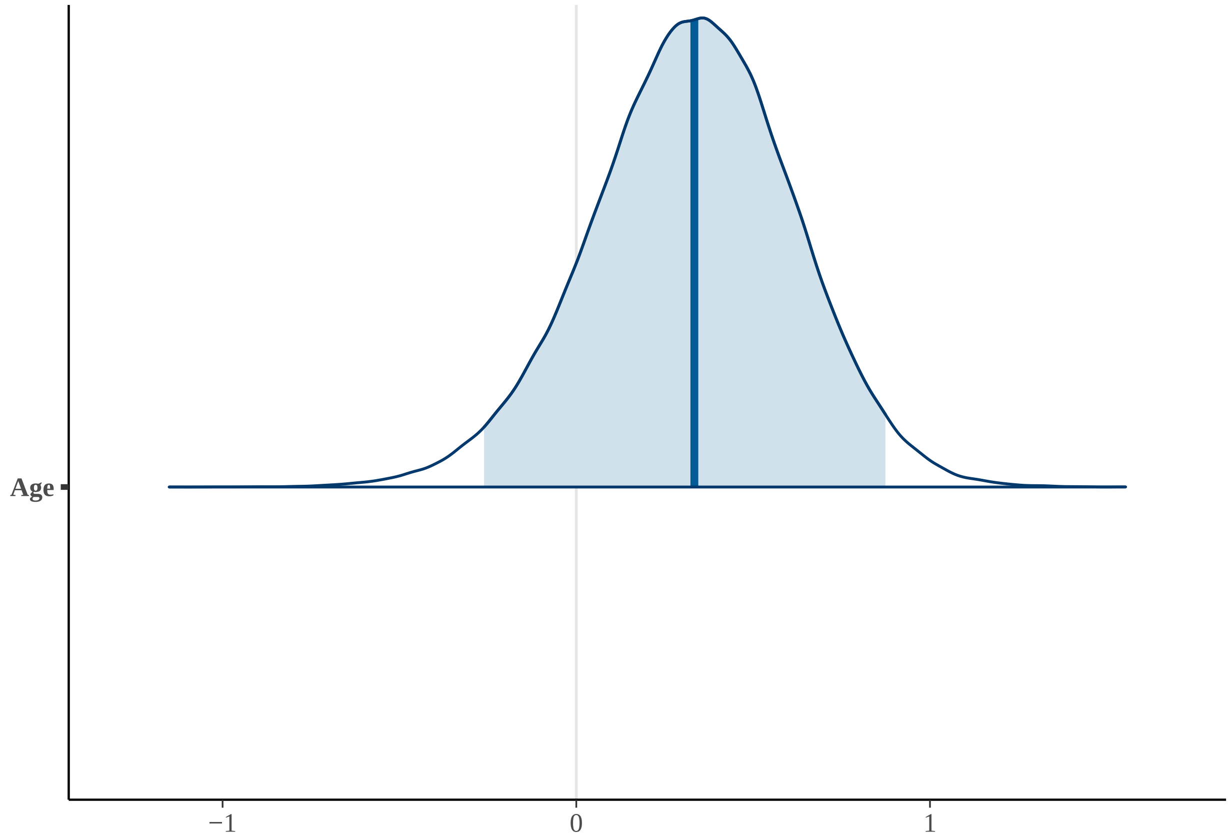

Figure S4: Posterior distribution of the living shoreline age coefficient from the model including mussels only in the low marsh.

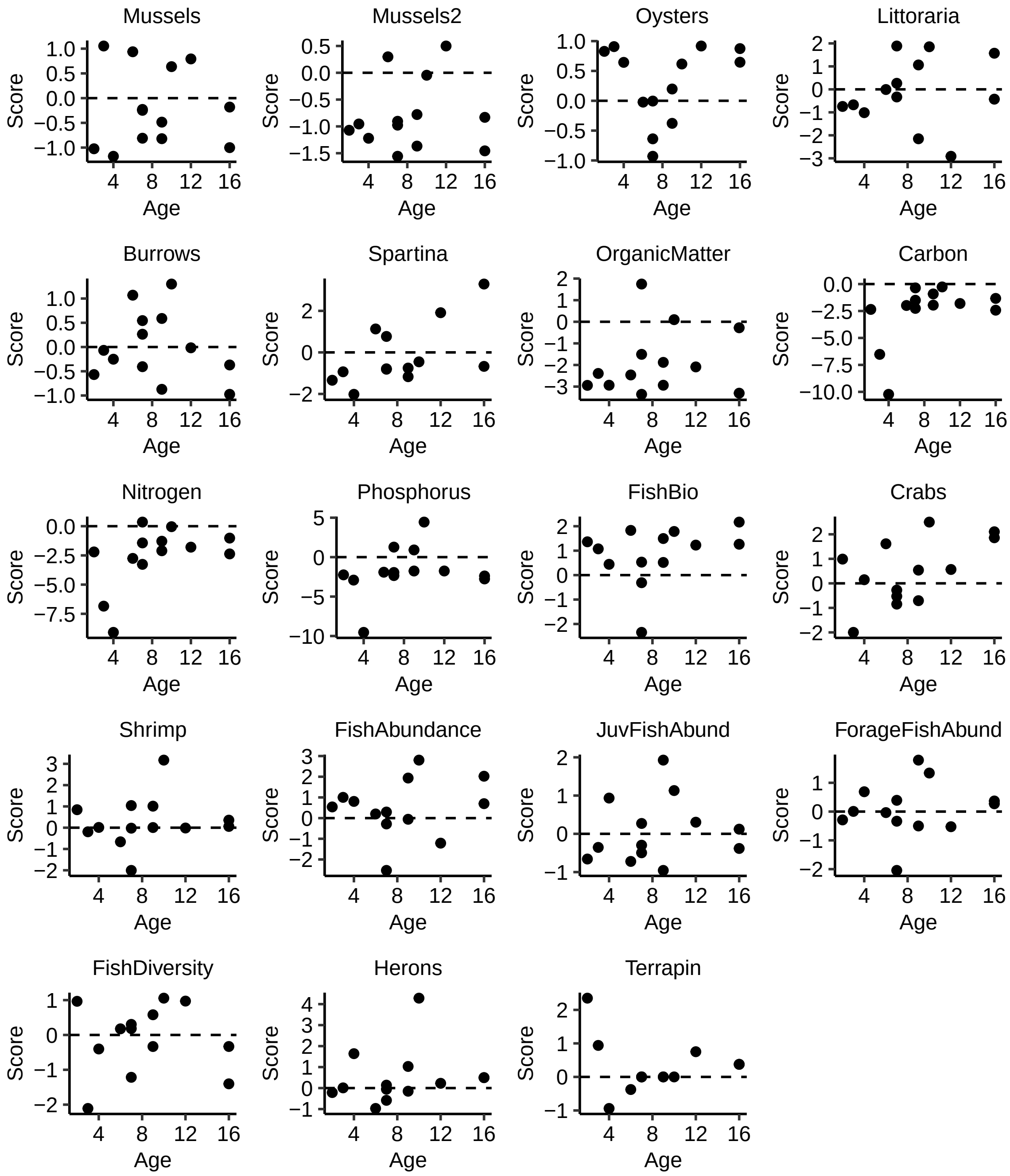

Figure S5: Z-score vs. living shoreline age (c. 2018) for each measured metric. Mussels2 corresponds to mussel density only in the low marsh.

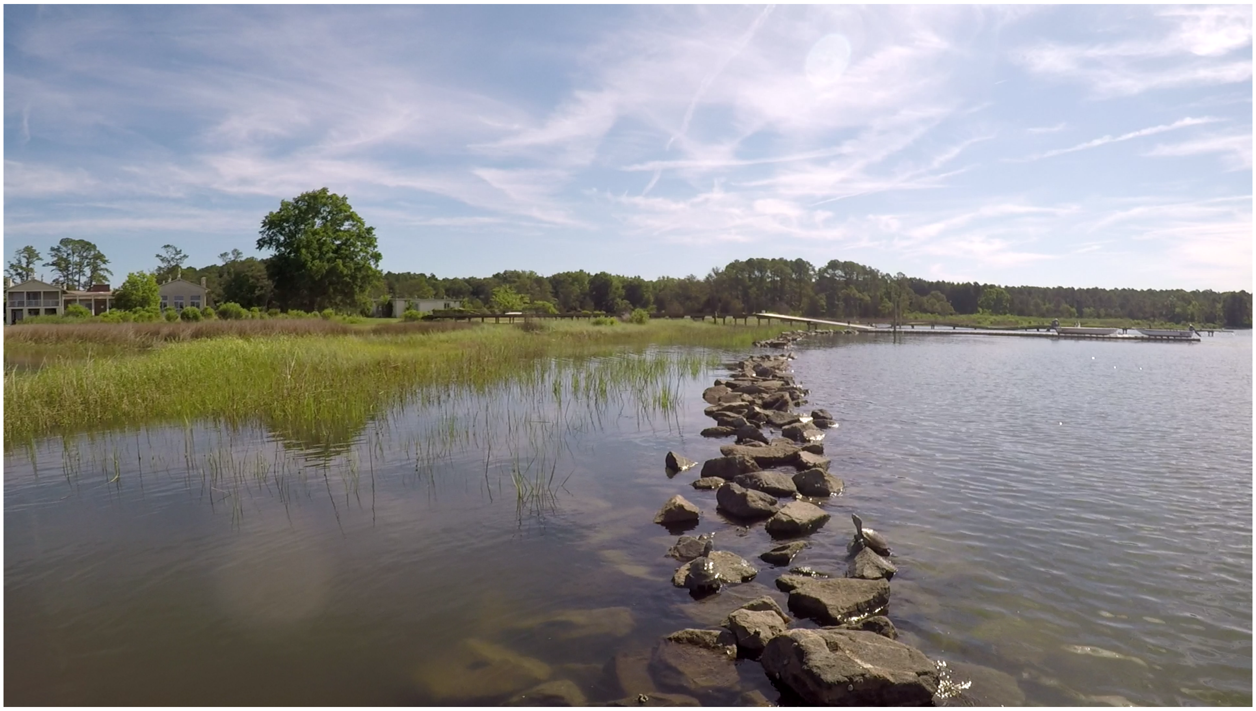

Figure S6: Diamondback terrapins basking on a living shoreline. Two diamondback terrapins can be seen basking on the living shoreline at one of the sites in the study.

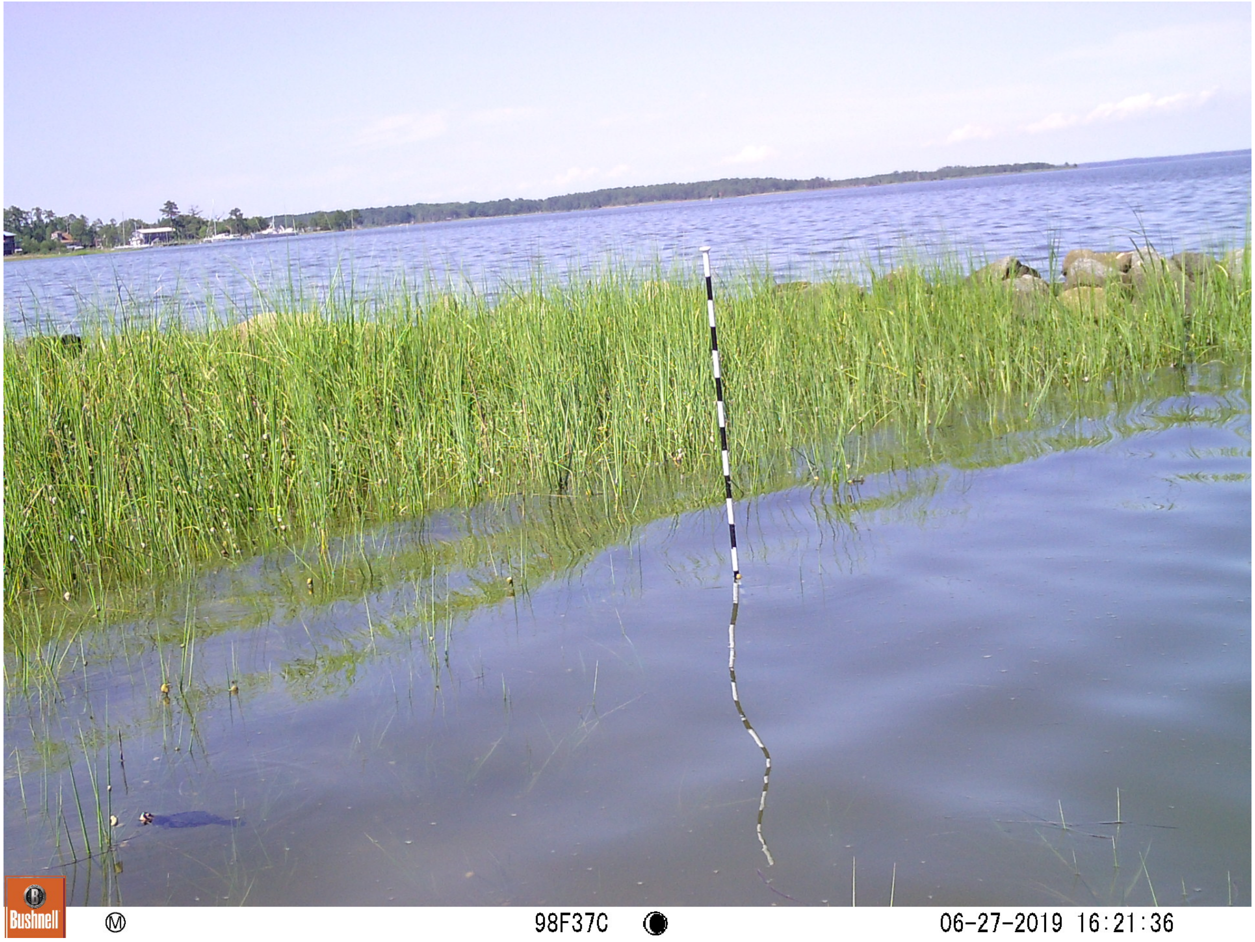

Figure S7: Diamondback terrapin swimming behind a living shoreline sill. A diamondback terrapin can be seen swimming behind the sill of the living shoreline.
